# Supplementary material for: Sensing Magnetic Field and Intermolecular Interactions in Diamagnetic Solution Using Residual Dipolar Couplings of Zephycandidine
Source: Int J Mol Sci. 2022 Dec 1;23(23):15118. doi: 10.3390/ijms232315118 (PMC9737189; doi:10.3390/ijms232315118)
Supplement: Supplementary file 1 [file ijms-23-15118-s001.zip › ijms-2064990-supplementary.pdf]

Table S1. Comparison of solvent properties and their deuterated equivalents. The values given in bold were selected for the analyses described in the manuscript.

	Solvent	Molar volume	Boiling point	*Refractive index*	*Dielectric constant*	Electric dipole moment		Volume magnetic susceptibility in air at normal pressure	Volume magnetic susceptibility
		MV	t_{BP}	n_D^{20}	ε	ρ		χ_{sol}	χ_{sol}
		cm^3/mol	°C			Cm	Debye	ppm	ppm
aromatic	benzene	89.4	80.1	1.5011	2.27	0.0 $\times 10^{-30}$	**0.000**		
	benzene-d_6	**88.6**	79.1	**1.4986**	2.3			**-7.37**	-7.56
	toluene	106.8	110.6	1.4969	2.38	1.0 $\times 10^{-30}$	**0.300**		
	toluene-d_8	**106.6**	109.0	**1.493**	2.4			**-7.90**	-8.01
protic	acetic acid	57.1	117.9	1.3719	6.17	5.6 $\times 10^{-30}$	**1.679**		**-6.66**
	acetic acid-d_4	**57.3**	115.5	**1.368**	6.1				
	methanol	40.7	64.5	1.3284	32.66	5.9 $\times 10^{-30}$	**1.769**		
	methanol-d_4	**40.5**	65.0	**1.3256**	32.7			**-6.69**	-6.79
	ethanol	58.5	78.3	1.3614	24.55	5.8 $\times 10^{-30}$	**1.739**		
	ethanol-d_6	**58.7**	78.0	**1.358**	24.5				**-7.29**
aprotic	dimethyl sulfoxide	71.3	189.0	1.4793	46.45	13.5 $\times 10^{-30}$	**4.047**		
	dimethyl sulfoxide-d_6	**70.7**	190.0	**1.4758**	46.7			**-7.80**	-7.83
	acetone	74.0	56.1	1.3587	20.56	9.0 $\times 10^{-30}$	**2.698**		
	acetone-d_6	**73.5**	55.5	**1.3554**	20.7			**-5.70**	-5.78
	tetrahydrofuran	81.7	66.0	1.4072	7.58	5.8 $\times 10^{-30}$	**1.739**		
	tetrahydrofuran-d_8	**81.0**	64.0	**1.4035**	7.6			**-7.96**	-8.08
	acetonitrile	52.6	81.6	1.3441	35.94	13.0 $\times 10^{-30}$	**3.897**		
	acetonitrile-d_3	**52.2**	80.7	**1.3406**	37.5			**-6.65**	-6.76
	chloroform	80.7	61.2	1.4459	4.89	3.8 $\times 10^{-30}$	**1.466**		
	chloroform-d	**80.3**	60.9	1.4445	4.8			**-9.24**	-9.07
	Data references:	protonated solvents - [48]; deuterated solvents - [63]	protonated solvents - [62]; deuterated solvents - [63]					deuterated solvents - [38]; acetic acid - [59, 60]; ethanol - [61]	

Units conversion for Electric dipole moment: $\boldsymbol{\rho}$ [C m] = 3.336×10^{-30} * $\boldsymbol{\rho}$ [Debye]

Table S2. Comparison of both experimental and estimated values of cohesive energy density (ced) from various literature sources. The values given in bold were selected for the analyses described in the manuscript.

		experimental		experimental		experimental		experimental		literature average		empirical estimated with some experimental confirmation			
Reference		[33]		[39]		[44]		[45]		[43]		[48]			
												Total	Polar	Dispersion	Hydrogen Bond
		cal cm^{-3}	J cm^{-3}	cal cm^{-3}	J cm^{-3}	cal cm^{-3}	J cm^{-3}	kBar	J cm^{-3}	cal cm^{-3}	J cm^{-3}	J cm^{-3}	J cm^{-3}	J cm^{-3}	J cm^{-3}
aromatic	benzene	83.7	350	85.0	**356**					83.7	350	343	***0***	***339***	***4***
	toluene	79.4	332	79.2	**331**	78.9	330			79.9	334	330	***2***	***324***	***4***
protic	acetic acid			102.0	**427**					107.1	448	457	***64***	***210***	***182***
	methanol	208.8	874	210.3	**880**	211.7	886			204.5	856	877	***151***	***228***	***497***
	ethanol	161.3	675	161.3	**675**					166.9	698	703	***77***	***250***	***376***
aprotic	dimethyl sulfoxide	168.6	705	144.0	**603**					210.3	880	712	***269***	***339***	***104***
	acetone	94.3	395	98.0	**410**	95.8	401			95.5	399	397	***108***	***240***	***49***
	tetrahydrofuran	86.9	364	82.8	**346**	87.4	366			82.8	347	379	***282***	***32***	***64***
	acetonitrile	139.2	582	141.6	**593**					142.1	595	595	***324***	***234***	***37***
	chloroform	85.4	357	86.5	**362**			3.61	361			359	***10***	***317***	***32***

Units conversion for ced: **ced** [J cm^{-3}] = **ced** [MPa] = 4.1840 ***ced** [cal cm^{-3}] = 4.1840 * $\boldsymbol{\delta}^2$ [$cal^{0.5}cm^{-1.5}$] = 100 ***ced** [kBar]

ced [MPa] = ($\boldsymbol{\delta}_D^2$ [$MPa^{0.5}$] + $\boldsymbol{\delta}_P^2$ [$MPa^{0.5}$] + $\boldsymbol{\delta}_H^2$ [$MPa^{0.5}$])

Table S3. The values of ced for acetic acid from various sources.

	ced	ced	technique	enthalpy	process	Reference
	cal cm^{-3}	J cm^{-3}		kJ/mol		
experimental	102.0	427	vapor pressure			[39]
literature average	107.1	448				[43]
empirical estimate		457				[48]
from experimental enthalpy of vaporization	165.2	691	vapor pressure	42.0		[47]
	87.2	365	calorimetric measurements	23.4	liquid to gas at equilibrium	[46]
	205.1	858	calorimetric measurements	51.6	liquid to monomer gas	[46]

Estimate of ced from enthalpy of vaporization:

ced [J cm^{-3}] = 4.1840 ***ced** [cal cm^{-3}] = $(\Delta H_{vap}$ [J mol^{-1}] - **R** [J mol^{-1} K^{-1}] **T** [K]$)$ * **MV**$^{-1}$ [cm^{-3} mol^{-1}]

Figure S1. (a) Temperature dependence of the quadrupolar splitting in the deuterium spectrum of benzene-d_6. (b) The 13C satellite signals in the deuterium spectrum of benzene-d_6 used to estimate quadrupolar splitting in (a). Red dotted lines represent simulations.

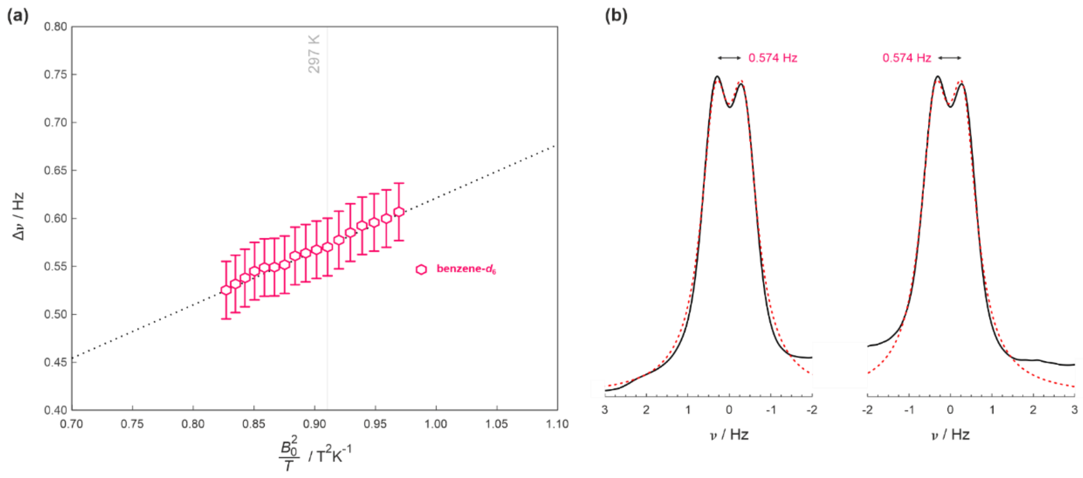

<u>The detailed step-by-step description of the procedure to estimate energy barrier:</u>

Step 1. From the magnetic field dependence (equation (2) in the manuscript) at constant room temperature (i.e., 297K), the value of the magnetic anisotropy $\Delta\chi_{ax}$ for each solvent is known (listed in Table 1 of the manuscript). Therefore, at a constant magnetic field of 16.445 T (700 MHz NMR spectrometer), the “predicted” splitting Δυ can be calculated for other temperatures at that magnetic field from the same equation (2).

Step 2. The natural logarithm of “predicted” splitting ln(Δυ) as function of the inverse of temperature (T^{-1}) can be fitted to the Arrhenius equation (dotted lines in Figure 5(b) in the manuscript). From the fit the energy required for the reorientation in the given solvent can be extracted.

Step 3. The natural logarithm of the experimentally measured (at 700 MHz) splitting ln(Δυ) as function of the inverse of temperature (T^{-1}) can be fitted to the Arrhenius equation (experimental points in Figure 5(b) in the manuscript). From the fit the additional energy required for reorientation can be extracted.

Step 4. The difference in the energies above is called the energy barrier Δ*E*.

References:

[33] Dack, M.R.J. The importance of solvent internal pressure and cohesion to solution phenomena. Chem. Soc. Rev. 1975, 4, 211-229.

[38] Hoffman, R.E. Magnetic susceptibility measurement by NMR: 2. The magnetic susceptibility of NMR solvents and their chemical shifts. J. Magn. Reson. 2022, 335, 107105.

[39] Barton, A.F.M Solubility parameters, Chem. Rev 1975, 75, 731-753.

[43] Belmares, M.; Blanco, M.; Goddard III, W.A.; Ross, R.B.; Caldwell, G.; Chou, S.-H.; Pham, J.; Olofson, P.M.; Thomas, C. Hildebrand and Hansen Solubility Parameters from Molecular dynamics with application to electronic nose polymer sensors. J. Comput. Chem. 2004, 25, 1814-1826

[44] D'Amelia, R.P.; Tomic, J.C.; Nirode, W.F. The determination of the solubility parameter (δ) and the Mark-Houwink constants (K & α) of food grade polyvinyl acetate (PVAc). J. Polym. Biopolym. Phys. Chem. 2014, 2, 67-72.

[45] Hübel, H.; Faux, D.A.; Jones, R.B.; Dunstan, D.J. Solvation pressure in chloroform. J. Chem. Phys. 2006, 124, 204506-1 - 204506-7.

[46] Konicek, J.; I. Wadsö, I. Enthalpies of vaporization of organic compounds, Acta Chem. Scand. 1970, 24, 2612-2616.

[47] Chickos, J.S.; Acree Jr., W.E. Enthalpies of vaporization of organic and organometallic compounds 1880-2002. J. Phys. Chem. Ref. Data 2003, 32, 519 – 878.

[48] Hansen, C.M. Hansen solubility Parameters. A user's handbook, 2nd ed.; Publisher: CRC Press, London New York, 2007; pp. 4–6, 13-17, 45-59.

[59] Deshpande, V.T.; Pathki, K.G. Magnetic susceptibility of acetic acid + pyridine mixtures. *Trans. Faraday Soc*. 1962, *58,* 2134-2138.

[60] Sriraman, S.; Shanmugasundaram, V.; Sabesan, R.; The diamagnetic study of the nature of the association of acetic acid. II. The binary systems of acetic acid with Triethylamine, pyridine and Aniline. Bull. Chem. Soc. Jpn. 1965, 38, 1391-1393.

[61] https://nmr.chem.umn.edu/ResReports/NMR002.htms

[62] Christian Reichardt C. and Welton T. Solvents and Solvent Effects in Organic Chemistry, 4th ed.; Willey: Weinheim, Germany, 2011; Appendix A, pp 549-553.

[63] Bruker Almanac 2012; NMR tables p15.
